# Effects of Lactic Acid Bacteria on Fermentation and Nutritional Value of BRS Capiaçu Elephant Grass Silage at Two Regrowth Ages

**DOI:** 10.3390/ani15081150

**Published:** 2025-04-17

**Authors:** Daiana Lopes Lelis, Mirton José Frota Morenz, Domingos Sávio Campos Paciullo, João Paulo Santos Roseira, Carlos Augusto de Miranda Gomide, Odilon Gomes Pereira, Jackson Silva e Oliveira, Fernando Cesar Ferraz Lopes, Vanessa Paula da Silva, Tâmara Chagas da Silveira, Fernanda Helena Martins Chizzotti

**Affiliations:** 1 Department of Animal Science, Federal University of Vicosa, Vicosa 36570-900, Brazil; dailopes04@gmail.com (D.L.L.); jpr-santos@hotmail.com (J.P.S.R.); odilon@ufv.br (O.G.P.); vanessapaula_s@yahoo.com (V.P.d.S.); tandacs@gmail.com (T.C.d.S.); 2 Embrapa Dairy Cattle, Brazilian Agricultural Research Corporation, Juiz de Fora 36038-330, Brazil; mirton.morenz@embrapa.br (M.J.F.M.); domingos.paciullo@embrapa.br (D.S.C.P.); carlos.gomide@embrapa.br (C.A.d.M.G.); jackson.oliveira@embrapa.br (J.S.e.O.); fernando.lopes@embrapa.br (F.C.F.L.)

**Keywords:** ammonia nitrogen, bacterial inoculant, tropical grass

## Abstract

The objective of this study was to evaluate the effects of lactic acid bacteria inoculation on the fermentation profile and nutritional value of BRS Capiaçu elephant grass silages harvested at two regrowth ages. The treatments were arranged in a 5 × 2 factorial scheme, with five inoculants (I) and two regrowth ages (A, 90 and 105 days), in a completely randomized design, with three replicates. There were I × A interactions (*p* < 0.05) on pH, acetic acid, and water-soluble carbohydrates. The silage treated with Kera-Sil showed a lower pH compared with the control silage. The highest ammonia nitrogen content was recorded in the silage treated with Yakult^®^. There were I × A interactions (*p* < 0.05) on the dry matter (DM) content, neutral detergent fiber (NDF), and in vitro digestibility of DM (IVDMD) and NDF (IVNDFD). Silages treated with Kera-Sil and Silo-Max at 90 days of regrowth showed a higher DM and higher IVDMD (*p* < 0.05). A higher NDF content and lower IVDMD and IVNDFD were recorded in silages produced with grass harvested at 105 days of regrowth (*p* < 0.05). The use of commercial microbial inoculants improved the fermentative and nutritional parameters of the silages.

## Simple Summary

Ensiling is a widely used technique for preserving forage in ruminant production systems worldwide. In tropical climates, elephant grass stands out as a crop for silage production due to its high productivity. During the ensiling process, losses are inevitable; however, they can be minimized through proper crop management and the use of additives such as bacterial inoculants. Adopting these practices enables the production of higher-quality silage, which can positively impact animal performance.

## 1. Introduction

Silage is the primary source of roughage in the diet of ruminants in semi-confined and confined production systems [1,2]. Elephant grass (*Cenchrus purpureus*) is a commonly grown forage material in tropical and subtropical regions [3]. In Brazil, the BRS Capiaçu cultivar stands out for its high productivity (50 tons dry matter (DM) ha/year) and its flexibility of use, both for fresh consumption and for silage production [4].

The use of strategies that favor the ensilage process is essential to ensure adequate conditions for the fermentation and conservation of ensiled biomass. In this context, the use of lactic acid bacteria (LAB) plays a fundamental role in modulating fermentation. Obligate homofermentative LAB ferments glucose, producing almost exclusively lactic acid (LA), while facultative heterofermentative LAB ferments both glucose and pentoses, producing primarily lactic and acetic acids [5]. In this way, they can promote a rapid reduction in the pH of the medium, due to the increase in the concentration of lactic acid in inoculated silages compared with untreated silage [6,7,8]. These conditions help reduce the occurrence of secondary fermentations, which cause DM losses and compromise the final quality of the silage [7,9]. However, the results on the use of inoculant in the ensilage of tropical grasses are inconsistent [10,11]. These variations can be explained by factors such as the availability of substrate in the environment and the relationship between the population of inoculated bacteria and the epiphytic population of the forage [12,13]. Other factors include the bacterial species and the interaction of the strain used with the forage crop [5,14,15], in addition to the moisture content of the forage plant at the time of harvest [16].

In search of cost reduction in the ensilage process, small producers use alternative inoculants, for example, fermented milk (Yakult^®^, Yakult Honsha, Tokyo, Japan), to replace commercial inoculants. This probiotic drink, composed of *Lacticaseibacillus casei*, has aroused the interest of many producers and technicians regarding its use in elephant grass ensilage. *Lacticaseibacillus casei* is a facultative heterofermentative bacterium [5] capable of increasing lactic acid concentration, which promotes a rapid pH decline, helping in secondary fermentation control in the ensiled mass [17,18]. However, the strain present in the fermented beverage was selected as a probiotic for humans [19], and its choice for use as an inoculant was not based on tests commonly used for screening lactic acid bacteria intended for silage [20]. Sriagtula et al. [21] did not observe any effects of the fermented beverage on the fermentative and nutritional characteristics of sorghum silages.

Due to the great potential of using BRS Capiaçu elephant grass silage in different production systems and the lack of scientific information on the effects of adding fermented milk to its ensilage process, there is a need for evaluation. Therefore, this study aimed to determine the effects of adding commercial inoculants and fermented milk on the fermentative profile and nutritional value of BRS Capiaçu elephant grass silage harvested at different regrowth ages. We hypothesize that the addition of commercial microbial inoculants and fermented milk improves the fermentative profile and nutritional value of BRS Capiaçu elephant grass silage.

## 2. Materials and Methods

### 2.1. Location and Silage Preparation

**Figure 1:**
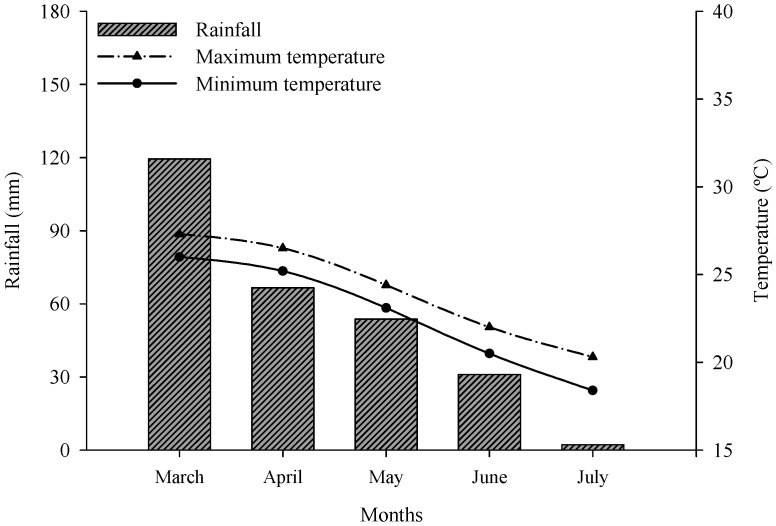
Rainfall (mm) and maximum and minimum temperatures (°C) during the experimental period.

This study was conducted at the Embrapa Dairy Cattle Farm in the city of Coronel Pacheco, Minas Gerais State, southeastern Brazil (**21°33′ S** and **43°06′ W**), at an average altitude of 410 m. According to the Köppen classification, the local climate is Cwa (mesothermal). During the cultivation of elephant grass for ensiling, climate data were collected from the automatic weather station of National Institute of Meteorology, located 300 m from the experimental area (Figure 1).

Soil correction, fertilization, and the establishment of BRS Capiaçu elephant grass were carried out according to the recommendations of Pereira et al. [4]. At planting, phosphate fertilizer (120 kg/ha of P_2_O_5_) was applied in furrows 20–30 cm deep, spaced 1 m apart. When the plants reached an average height of 50 cm, 1200 kg/ha of NPK **20-05-20** was applied. Sixteen months after the establishment of the area, a standardized cut was performed. After 90 and 105 days of regrowth, the elephant grass, with an average height of 2.40 ± 0.23 and 3.30 ± 0.06 m, respectively, was harvested manually at a height of 10 cm above the soil surface in an area of 27 m^2^. Then, it was weighed to estimate the forage production, and samples of nine tillers representative of the grass average height were sent to the laboratory for the separation of morphological components and determination of the leaf blade-to-stem ratio. Then, the elephant grass was chopped in a stationary forage machine (model EN-9F3B, Nogueira^®^ S.A., São João da Boa Vista, SP, Brazil) to an average particle size of 1 cm.

Three piles (replicates) were prepared, containing 1.8 kg of forage for each inoculant, for a total of 15 piles per regrowth day. They were inoculated individually as follows: without inoculant (control, only distilled water); with Kera-Sil (Kera Nutrição Animal^®^, Bento Gonçalves, Brazil), composed of *Lactiplantibacillus plantarum* and *Pediococcus acidilactici*; with Sil-All 4 × 4 W.S (Lallemand^®^, Aparecida de Goiania, Brazil), composed of *L. plantarum*, *P. acidilactici*, *Enterococcus faecium*, *Ligilactobacillus salivarius*, xylanase, amylase, cellulase, hemicellulase, silicon dioxide, and sucrose; with Silo-Max Centurium (Matsuda^®^, Álvares Machado, Brazil), composed of *L. plantarum*, *L. lactis*, and *P. acidilactici*; and with fermented milk (Yakult^®^, Yakult Honsha, Tokyo, Japan), composed of *L. casei*, skimmed milk and/or reconstituted skimmed milk, maltitol syrup, polydextrose, glucose, lactic ferment, pectin stabilizer, flavoring, and sucralose (a sweetener). The recommended dosage of the commercial inoculant forage was applied as per the manufacturer’s instructions. For the Yakult^®^ fermented milk, the adopted recommendation followed the average that producers usually use in silos on their properties: a dilution of 8 mL of fermented milk in 1 L of distilled water applied at 10 L/ton of forage, based on natural material (NM). After homogenization, the forage was manually ensiled at an average density of 570 kg NM/m^3^ in mini polyvinyl chloride (PVC) silos (40 cm high and 10 cm in diameter) equipped with a Bunsen valve to release gases. The silos were sealed and stored for 60 days in a protected location at room temperature.

A 5 × 2 factorial scheme was used, with five inoculants (I), namely, control, Kera-Sil, Sil-All, Silo-Max, and Yakult, and two regrowth ages (A, 90 and 105 days), in a completely randomized design, with three replications.

### 2.2. Fermentation Profile

An aqueous extract was prepared by combining 25 g of the plant or silage with 225 mL of saline solution (Ringer Solution, Oxoid^®^, Basingstoke, UK) and homogenizing for 1 min in an industrial blender. A 10-mL aliquot of the extract was filtered through sterile gauze, acidified with 50% H_2_SO_4_, and stored at −20 °C for subsequent analysis of water-soluble carbohydrates (WSC) according to Nelson [22].

The pH of the plant or silage was measured using a potentiometer (Tecnal^®^, São Paulo, Brazil) in an extract produced after hydraulic pressing of the plant or silage. A 10-mL aliquot the silage extract was collected and acidified with 25% metaphosphoric acid to determine the ammonia nitrogen (NH_3_-N) [23], LA, acetic acid (AA), propionic acid (PA), and butyric acid (BA) [24] contents. High-performance liquid chromatography (HPLC) was used to analyze the organic acid contents, and the instrument was equipped with a PAD 2998 photodiode array detector and a separation system comprising a C18 ODS 80A reversed-phase column (150 mm × 4.6 mm × 5 μm). Samples were analyzed in duplicate.

### 2.3. Chemical Composition and In Vitro Digestibility

Forage samples prior to ensiling and silages were dried in a forced ventilation oven at 55 °C for 72 h and then ground in a Willey mill with a 1-mm sieve. The DM (INCT-CA G-003/1 method), ash (INCT-CA M-001/1 method), crude protein (CP; INCT-CA N-001/1 method), neutral detergent insoluble fiber corrected for ash and protein (NDFap; INCT-CA F-002/1 method), acid detergent insoluble nitrogen (ADIN; INCT-CA N-005/1 method), and lignin (INCT-CA F-005/1 method) contents were analyzed as described previously [25].

The in vitro digestibility of DM (IVDMD) and neutral detergent fiber (IVNDFD) were estimated using an ANKOM incubator (Ankom^®^ Technology Corporation, Fairport, NY, USA), and following the method proposed by Tilley and Terry [26] and adapted by Holden [27]. The inoculum was collected from three rumen-fistulated lactating cows fed a diet based on elephant grass silage (60%) and concentrate (40%), on a DM basis.

### 2.4. Statistical Analysis

The data were submitted to Shapiro–Wilk test to determine whether the residuals followed a normal distribution (*p* < 0.10). The data were analyzed in a factorial scheme in a completely randomized design. The inoculants, the regrowth age, and the interaction between these two factors were considered fixed effects, according to the following model:

Y*_ijk_* = μ + I*_i_* + A*_j_* + (IA)*_ij_* + e*_ijk_*,

where Y*_ijk_* is the response variable; μ is a general constant; I*_i_* is the effect of inoculant *i*; A*_j_* is the effect of regrowth age *j*; (IA)*_ij_* is the interaction of inoculant *i* and regrowth age *j*; and e*_ijk_* is a random error assuming an independent normal distribution (0, σ^2^). After analysis of variance, the significant interactions among the factors were assessed with Fisher’s minimum significant difference using the PDIFF option of the LSMEANS command for the regrowth age and the Tukey test for inoculants. A critical probability level of 0.05 was adopted for a type I error, using PROC MIXED in SAS version 9.4 [28].

## 3. Results

### 3.1. Forage Characterization

The agronomic characteristics and nutritional value of BRS Capiaçu elephant grass before ensiling are presented in Table 1. The agronomic data indicate numerically higher production of green mass and DM for grass harvested at 105 days, but with a lower leaf blade-to-stem ratio compared with a regrowth age of 90 days. DM, NDFap, lignin, ADIN, and WSC were numerically higher at 105 days of regrowth, while the ash, CP, IVDMD, and IVNDFD were higher at 90 days of regrowth.

### 3.2. Fermentation Profile

The I × A interaction was significant (*p* < 0.05) on pH, AA, the LA-to-AA ratio, and the residual WSC, while NH_3_-N, LA, and PA were only affected by the I (*p* < 0.05) (Table 2).

There was higher residual WSC (6.24 g/kg DM) in the silages treated with Kera-Sil for both regrowth ages (*p* < 0.05) compared with the other silages and WSC at 105 days of regrowth compared with 90 days of regrowth (9.34 vs. 6.24 g/kg DM, *p* < 0.05) (Table 2).

The silage inoculated with Kera-Sil presented a lower pH (*p* < 0.05) compared with the control silage and the silages inoculated with Sil-All and Yakult when the grass was harvested at 90 days of regrowth. At 105 days of regrowth, the silages inoculated with Kera-Sil and Sil-All presented a lower pH (*p* < 0.05) compared with the control silage and the silages treated with Silo-Max. There were no differences in the pH (*p* > 0.05) of silages produced with grass with different regrowth ages for any of the inoculants (Table 2).

Lactic acid was similar for the control and inoculated silages (*p* > 0.05). However, LA was higher for the silage treated with Sil-All compared with the silage treated with Silo-Max (34.44 g/kg DM vs. 23.22 g/kg DM, *p* < 0.05).

Acetic acid was lower in the silages inoculated with Kera-Sil compared with the control silages at 90 and 105 days of regrowth (*p* < 0.05), which resulted in a higher LA-to-AA ratio in these silages compared with the others. However, AA was similar in the silages produced with grass harvested at 90 and 105 days of regrowth (*p* > 0.05). The control silage and the silage treated with Silo-Max had a higher LA-to-AA ratios when produced with grass harvested at 90 days of regrowth compared with 105 days of regrowth (Table 2).

Propionic acid was higher in the silage treated with Sil-All compared with the silage treated with Silo-Max (*p* < 0.05). However, PA was similar in the inoculated and control silages (*p* > 0.05). PA was higher when using a regrowth age of 90 days compared with 105 days (4.22 vs. 3.40 g/kg DM) (Table 2). Butyric acid was not detected in the silages examined in the present study.

NH_3_-N was highest in the silage treated with Yakult^®^ (11.18% total nitrogen (TN)) and was significantly higher compared with the other silages (*p* < 0.001), while the silage treated with Kera-Sil had the lowest NH_3_-N (5.77% TN). NH_3_-N was not influenced by the grass regrowth age (*p* = 0.639), with an average of 8.47% TN (Table 2).

### 3.3. Chemical Composition and In Vitro Digestibility

There was a significant I × A interaction on DM (*p* < 0.001) and NDFap (*p* = 0.045) (Table 3). DM was higher in the silages using grass with 90 days of regrowth and treated with Kera-Sil and Silo-Max compared with the others (*p* < 0.05). However, when using grass with 105 days of regrowth, there was no inoculant effect (*p* > 0.05). DM was higher in the control silage and the silages treated with Sil-All and Yakult^®^ when using grass with 105 days of regrowth compared with grass with 90 days of regrowth (*p* < 0.05).

At 90 and 105 days of regrowth, NDFap was lower in the silages treated with Kera-Sil compared with the silages treated with Yakult^®^ and Silo-Max, respectively (*p* < 0.05). However, at both regrowth ages, NDFap was similar in the inoculated and control silages (*p* > 0.05). NDFap was higher in the silages with 105 days of grass regrowth compared with 90 days of regrowth (*p* < 0.05) (Table 3).

Crude protein was only affected by the regrowth age (*p* < 0.001), with a higher content in the silages with 90 days of regrowth compared with 105 days of regrowth (67.04 vs. 50.15 g/kg DM).

Lignin was affected by the I (*p* = 0.010) and A (*p* = 0.001). Lignin was higher in the silage treated with Sil-All compared with the silage treated with Silo-Max (66.40 vs. 53.94 g/kg DM). Moreover, lignin was higher in the silages with a grass regrowth age of 105 days compared with 90 days (68.14 vs. 50.95 g/kg DM).

Regarding ADIN, the regrowth age had a significant effect (*p* = 0.002): It was higher in the silages using grass with 105 days of regrowth compared with 90 days of regrowth (68.67 vs. 53.01 g/kg TN). The inoculants did not have an effect on ADIN (*p* = 0.302) with a mean value of 60.84 g/kg TN.

There was an I × A interaction on IVDMD (*p* = 0.005) and IVNDFD (*p* = 0.009) (Table 3). The silages produced with grass harvested at 90 days of regrowth and treated with Kera-Sil and Silo-Max presented higher IVDMD compared with the control silage (*p* < 0.05). At 105 days of regrowth, only the silage treated with Kera-Sil showed a higher IVDMD than the control silage. Regarding IVNDFD, the silage using grass with 90 days of regrowth and treated with Silo-Max was superior compared with the control silage and those treated with Sil-All and Yakult^®^ (*p* < 0.05). For the silages using grass with 105 days of regrowth, the inoculants did not have an effect (*p* > 0.05). IVDMD and IVNDFD were higher for all treatments when the silages were produced with grass harvested at 90 days of regrowth compared with 105 days of regrowth (Table 3).

## 4. Discussion

The numerical differences observed for the leaf blade-to-stem ratio, CP contents, cell wall constituents, and digestibility before ensiling indicated that the nutritional quality of elephant grass decreases with maturity, consistent with what has been reported in previous studies [29,30,31]. However, we noted an increase in DM productivity as well as the DM content of the forage, which is an important characteristic for the fermentation process of the ensiled mass [31,32].

The WSC contents of elephant grass before ensiling were in accordance with the recommendation of McDonald et al. [32], who established a value of 60–80 g/kg DM as ideal to optimize LAB metabolism and to promote a rapid decline in the pH of the ensiled mass. The pH, which ranged from 3.70 to 4.16, was adequate for the silages evaluated in the present study and are lower than those reported by Kung et al. [33], who reported a pH of 4.3–4.7 for grass silages. A higher WSC content is also desirable at the time of silo opening: It reflects good fermentation of the ensiled mass, resulting in greater availability of energy-producing substrates for ruminal microorganisms [34]. In our study, there were higher residual WSC contents in the silages treated with Kera-Sil, possibly due to faster stabilization of the silage pH, leading to a shorter fermentation period and greater conservation of WSC. Kung et al. [33] highlighted that a low pH stabilizes silage fermentation by inhibiting the growth of or killing microorganisms intolerant of a low pH.

Lactic acid (pKa = 3.86) is widely recognized as being highly effective in reducing pH during fermentation [33]. Although the LA contents did not differ significantly between the control silage and the silages inoculated with Kera-Sil and Sil-All, the latter presented numerically higher LA contents compared with the control silage. This increase may have contributed to the lower pH observed in these silages at 90 and 105 days of regrowth when inoculated with Kera-Sil, and at 105 days of regrowth when inoculated with Sil-All. It is important to emphasize that, although the commercial inoculants have the species *L. plantarum* and *P. acidilactici* in common, there may be intraspecific variability between the strains, which can result in different responses [15,35].

Gandra et al. [36] reported that *P. acidilactici* strains predominate and thus guarantee a reduction in pH at the beginning of fermentation, even when the initial pH is high. Thereafter, when the pH starts to drop to around 5.0, *L. plantarum* produces LA, leading to a rapid drop in silage pH. Pahlow et al. [9] also noted that *P. acidilactici* grows vigorously at 15–50 °C and up to a pH of 3.6, with it potentially being effective in stimulating the *L. plantarum* population at later stages of ensiling.

The AA contents observed in this study, except for the control treatment at 105 days of regrowth, were below the minimum value of 10 g/kg DM reported by Kung et al. [33] for grass silages. Silages with a good fermentation pattern have an LA-to-AA ratio of 2.5–3.0. However, silages treated with LAB may show an increase in this proportion due to the almost exclusive production of LA [33], which probably occurred in the silage treated with Kera-Sil compared with the control silage. Moderate AA and PA contents in silage can be advantageous, as they help inhibit yeast growth, promoting greater stability when the silage is exposed to air [33,37]. The higher PA contents observed in silages produced with grass at 90 days of regrowth were probably related to the lower DM content of the grass at the time of ensiling. This factor favored the occurrence of secondary fermentations, possibly due to the metabolism of *Clostridium propionicum* [33]. Consistently, Silveira et al. [16] also recorded a higher PA content in elephant grass silage harvested at 8 weeks compared with that harvested at 16 weeks.

Facultative heterofermentative bacteria stand out for their rapid fermentation rate [5]. These bacteria predominantly produce LA, accelerating the pH decline and allowing the silage to reach the stability phase more quickly [5,6,38]. A rapid decline in pH inhibits the metabolism of bacteria of the genus *Clostridium*, which are associated with the degradation of CP and the consequent increase in NH_3_-N in the ensiled mass [16,32]. In the present study, the lower pH recorded in the silage inoculated with Kera-Sil probably contributed to the reduction in the growth of undesirable microorganisms, resulting in silage with a lower NH_3_-N content, as reported in previous studies [16,38,39]. However, the other silages evaluated presented an NH_3_-N content of 80–120 g/kg TN, which is considered acceptable for grass silages [33]. Therefore, we can infer that none of the silages showed relevant activity of bacteria of the genus *Clostridium*, which is capable of negatively affecting the fermentation profile and nutritional value of the forage. This can be proven by the CP contents of the silages, which remained close to the values observed in the forage before ensilage at both regrowth ages, as well as by the absence of BA in the silages we evaluated.

The DM content of the grass harvested at both regrowth ages was <250 g/kg of NM, which McDonald et al. [32] suggested is adequate for good fermentation of the ensiled biomass. Despite the high moisture content, all silages presented a good visual appearance, without any unpleasant odor or BA, as mentioned previously. The higher DM content in the silages inoculated with Kera-Sil and Silo-Max compared with the others, when the grass was harvested 90 days after regrowth, may have been related to lower losses during the fermentation process. Consistent with our findings, Ribas et al. [8] observed a higher DM content in BRS Capiaçu elephant grass silage treated with bacterial-enzymatic inoculants and attributed this result to a more pronounced reduction in pH.

The fact that the silages produced from grass harvested at 90 days had the highest CP contents and the lowest fibrous fraction contents reflects their greater nutritional value compared with those from grass harvested at 105 days. Indeed, younger plants have a lower proportion of fibrous and lignified tissues and a higher CP content [40]. However, these differences in the chemical composition of the grass at the two regrowth ages did not affect the fermentation profile of the silages, as there were no differences in the pH or NH_3_-N, LA, and AA contents depending on the regrowth age of the grass.

It is important to highlight that all silages presented an ADIN content below the minimum of 200 g/kg of TN, as proposed by Van Soest and Mason [41]. This result is desirable for ruminant nutrition, because this fraction is unavailable to ruminal microorganisms. The increase in ADIN levels indicates a lower digestibility of nitrogen, resulting in a reduction of amino acid absorption and potentially compromising the efficiency of forage use by animals [42]. In addition, a high ADIN content indicates excessive heating of the silage, which, in turn, is associated with the occurrence of secondary fermentations [33,43].

The higher IVDMD observed in the silages treated with Kera-Sil and Silo-Max compared with the control silage at 90 days after harvest was probably due to the lower lignin contents recorded in these silages, as well as lower losses during fermentation. Cai et al. [44] observed that IVDMD was improved in inoculated silages compared with a control. This effect was attributed to the action of LAB, which reduced DM losses during the silage fermentation process. Another possible explanation is the reduction of indigestible fiber fractions, resulting from the acid hydrolysis of hemicellulose caused by the reduction in the pH of the medium during fermentation by LAB [11,45]. The reductions in IVDMD and IVNDFD observed in the silages with increasing grass regrowth age were attributed to changes in the chemical composition of the plant, such as increased fiber content. This indicates a reduction in the nutritional value as the grass matures [29,30,40].

## 5. Conclusions

Silages produced with BRS Capiaçu elephant grass harvested at 90 or 105 days of regrowth showed an adequate fermentation profile, although there was a reduction in nutritional value with maturity. The use of commercial microbial inoculants improved some aspects of the fermentation profile and nutritional value of the silages. In particular, Kera-Sil resulted in silages with a lower pH and a lower NH_3_-N content, in addition to higher DM content and IVDMD. On the other hand, the use of Yakult^®^ did not improve the silage characteristics and, therefore, is not recommended for silage production with the BRS Capiaçu cultivar.

It is important to highlight that the adequate fermentation profile observed in the silages in this study, even without the application of inoculants, was probably related to the characteristics of the BRS Capiaçu elephant grass. Among them, the adequate content of WSC and the epiphytic population of LAB stand out, which favor efficient fermentation, combined with the correct execution of the ensiling process.

## Figures and Tables

**Table 1:** Agronomic characteristics and nutritive value of BRS Capiaçu elephant grass according to regrowth age.

Item ^1^	Regrowth Age (Days)	
	90	105
Green mass of forage (ton/ha)	36.41	51.37
Dry mass of forage (ton/ha DM)	6.77	10.79
Leaf blade-to-stem ratio	0.66 ± 0.13	0.38 ± 0.06
DM (g/kg NM)	186.11 ± 2.69	210.40 ± 1.47
Ash (g/kg DM)	121.40 ± 1.83	96.90 ± 1.48
CP (g/kg DM)	69.43 ± 1.24	53.95 ± 1.91
NDFap (g/kg DM)	663.40 ± 1.55	697.80 ± 0.96
Lignin (g/kg DM)	52.59 ± 1.73	72.56 ± 1.45
ADIN (g/kg of TN)	47.67 ± 1.55	64.46 ± 0.86
IVDMD (g/kg DM)	614.82 ± 3.66	586.81 ± 1.85
IVNDFD (g/kg DM)	418.60 ± 5.52	413.02 ± 2.62
WSC (g/kg DM)	71.77 ± 1.45	78.84 ± 1.56
pH	6.03 ± 0.01	6.05 ± 0.01

^1^ DM, dry matter; NM, natural matter; CP, crude protein; NDFap, neutral detergent fiber corrected for ash and protein; ADIN, acid detergent insoluble nitrogen; TN, total nitrogen; IVDMD, in vitro digestibility of DM; IVNDFD, in vitro digestibility of NDF; WSC, water-soluble carbohydrates. Values that follow the mean refer to the standard deviation.

**Table 2:** Means of residual water-soluble carbohydrates (g/kg DM), pH, organic acids (g/kg DM) and ammonia nitrogen (% TN, total nitrogen) of BRS Capiaçu elephant grass silages harvested at different ages and treated with different inoculants.

Regrowth Age (Days)	Inoculant	Item ^1^						
		WSC	pH	LA	AA	LA:AA	PA	NH_3_-N
90	Control	3.65 ^Ab^	3.98 ^Aa^	28.73	8.16 ^Aa^	3.50 ^Ab^	4.87	9.25
	Kera-Sil	6.24 ^Ba^	3.70 ^Ab^	25.87	3.18 ^Ab^	8.32 ^Aa^	3.57	4.88
	Sil-All	3.51 ^Ab^	3.91 ^Aa^	32.81	8.82 ^Aa^	3.71 ^Bb^	5.21	8.81
	Silo-Max	2.80 ^Ab^	3.85 ^Aab^	21.69	5.03 ^Aab^	4.24 ^Ab^	3.02	7.33
	Yakult	3.69 ^Ab^	3.97 ^Aa^	27.66	7.98 ^Aa^	3.54 ^Ab^	4.45	11.41
105	Control	3.02 ^Ab^	4.16 ^Aa^	25.15	11.09 ^Aa^	2.26 ^Bc^	3.31	8.68
	Kera-Sil	9.34 ^Aa^	3.81 ^Ab^	32.69	4.29 ^Ab^	7.59 ^Aa^	3.22	6.67
	Sil-All	3.95 ^Ab^	3.80 ^Ab^	36.08	7.47 ^Aab^	4.79 ^Ab^	4.43	8.03
	Silo-Max	2.79 ^Ab^	4.07 ^Aa^	24.76	8.23 ^Aa^	3.06 ^Bc^	2.93	8.67
	Yakult	2.96 ^Ab^	3.99 ^Aab^	28.46	9.02 ^Aa^	3.16 ^Ac^	4.20	10.96
SEM ^2^		0.2896	0.043	3.3582	0.7476	0.2103	0.4349	0.6075
Overall average for inoculant								
Control		3.34	4.07	26.94 ^ab^	9.63	2.88	4.09 ^ab^	8.96 ^b^
Kera-Sil		7.79	3.75	29.28 ^ab^	3.74	7.95	3.39 ^ab^	5.77 ^c^
Sil-All		3.73	3.86	34.44 ^a^	8.15	4.25	4.43 ^a^	8.42 ^b^
Silo-Max		2.79	3.96	23.22 ^b^	6.63	3.65	2.93 ^b^	7.99 ^b^
Yakult		3.33	3.98	28.06 ^ab^	8.50	3.35	4.20 ^ab^	11.18 ^a^
Overall average for regrowth age								
90		3.98	3.89	27.35	6.64	4.67	4.22 ^A^	8.34
105		4.42	3.97	29.43	8.02	4.18	3.40 ^B^	8.60
*p*-value ^3^								
I		<0.001	<0.001	0.053	<0.001	<0.001	0.017	<0.001
A		0.076	0.051	0.383	0.042	0.021	0.040	0.639
I × A		<0.001	0.008	0.635	0.050	0.003	0.344	0.090

^1^ WSC, water-soluble carbohydrates; LA, lactic acid; AA, acetic acid; LA:AA, lactic acid:acetic acid ratio; PA, propionic acid; NH_3_-N, ammonia nitrogen. ^2^ SEM, mean standard error. ^3^ I, inoculant; A, regrowth age; I × A, interaction. Different uppercase letters indicate a difference between the regrowth ages in each inoculant by PDIFF and different lowercase letters indicate a difference between inoculants in the respective ages based on the Tukey test (*p* < 0.05).

**Table 3:** Means of chemical composition (g/kg DM), in vitro digestibility of dry matter (IVDMD) and neutral detergent fiber (IVNDFD) (g/kg DM) of BRS Capiaçu elephant grass silages harvested at different ages and treated with different inoculants.

Item ^1^	Regrowth Age (Days)										SEM ^2^	*p*-Value ^3^		
	90					105						I	A	I × A
	Control	Kera-Sil	Sil-All	Silo-Max	Yakult	Control	Kera-Sil	Sil-All	Silo-Max	Yakult				
DM	163.77 ^Bb^	208.16 ^Aa^	153.21 ^Bb^	199.72 ^Aa^	153.02 ^Bb^	208.32 ^Aa^	205.58 ^Aa^	212.86 ^Aa^	213.36 ^Aa^	204.15 ^Aa^	5.046	<0.001	0.005	<0.001
CP	66.48	67.40	65.15	69.10	67.06	50.80	49.39	50.96	50.81	48.74	0.990	0.123	<0.001	0.065
NDFap	667.09 ^Bab^	652.19 ^Bb^	664.92 ^Bab^	660.00 ^Bab^	669.82 ^Ba^	695.54 ^Aab^	685.61 ^Ab^	697.39 ^Aab^	707.31 ^Aa^	700.51 ^Aab^	3.510	0.005	0.004	0.045
ADIN *	63.25	59.87	62.23	56.53	62.32	54.88	48.21	54.02	52.25	55.69	3.328	0.302	0.001	0.308
Lignin	55.19	46.19	59.02	47.15	47.21	69.99	67.79	73.78	60.73	68.40	3.206	0.010	0.001	0.583
IVDMD	677.64 ^Ac^	697.47 ^Aab^	685.03 ^Abc^	701.87 ^Aa^	671.91 ^Ac^	604.72 ^Bb^	626.28 ^Ba^	68.85 ^Bb^	603.86 ^Bb^	610.42 ^Bab^	3.171	<0.001	<0.001	0.005
IVNDFD	516.76 ^Abc^	535.96 ^Aab^	526.31 ^Abc^	548.34 ^Aa^	510.11 ^Ac^	431.68 ^Ba^	495.43 ^Ba^	439.11 ^Ba^	439.93 ^Ba^	443.78 ^Ba^	5.175	0.001	<0.001	0.009

^1^ DM, dry matter; CP, crude protein; NDFap, neutral detergent fiber corrected for ash and protein; ADIN, acid detergent insoluble nitrogen. ^2^ SEM, mean standard error. ^3^ I, inoculant; A, regrowth age; I × A, interaction. * Expressed in g/kg TN. Different uppercase letters on the line indicate a difference between the regrowth ages in each inoculant by PDIFF and different lowercase letters indicate a difference between inoculants in the respective ages based on the Tukey test (*p* < 0.05).

## Data Availability

All data generated or analyzed during this study are included in this published article.
